# Time-varying associations between prenatal metal mixtures and rapid visual processing in children

**DOI:** 10.1186/s12940-019-0526-y

**Published:** 2019-10-30

**Authors:** Yuri Levin-Schwartz, Chris Gennings, Lourdes Schnaas, María del Carmen Hernández Chávez, David C. Bellinger, Martha Maria Téllez-Rojo, Andrea A. Baccarelli, Robert O. Wright

**Affiliations:** 1 Department of Environmental Medicine and Public Health, Icahn School of Medicine at Mount Sinai, One Gustave L. Levy Place, Box 1057, New York, NY 10029 USA; 2 National Institute of Perinatology, Mexico City, Mexico; 3 Department of Neurology, Boston Children’s Hospital, Boston, MA USA; 4 National Institute of Public Health, Cuernavaca, Mexico; 5 Department of Environmental Health Sciences, Mailman School of Public Health Columbia University, New York, NY USA

**Keywords:** Heavy metals, Neurodevelopment, Mixtures, Childhood, Novel method

## Abstract

**Background:**

Humans are exposed to mixtures of chemicals across their lifetimes, a concept sometimes called the “exposome.” Mixtures likely have temporal “critical windows” of susceptibility like single agents and measuring them repeatedly might help to define such windows. Common approaches to evaluate the effects of chemical mixtures have focused on their effects at a single time point. Our goal is to expand upon these previous techniques and examine the time-varying critical windows for metal mixtures on subsequent neurobehavior in children.

**Methods:**

We propose two methods, joint weighted quantile sum regression (JWQS) and meta-weighted quantile sum regression (MWQS), to estimate the effects of chemical mixtures measured across multiple time points, *while providing data on their critical windows of exposure*. We compare the performance of both methods using simulations. We also applied both techniques to assess second and third trimester metal mixture effects in predicting performance in the Rapid Visual Processing (RVP) task from the *Cambridge Neuropsychological Test Automated Battery (CANTAB)* assessed at 6–9 years in children who are part of the PROGRESS (Programming Research in Obesity, GRowth, Environment and Social Stressors) longitudinal cohort study. The metals, arsenic, cadmium (Cd), cesium, chromium, lead (Pb) and antimony (Sb) were selected based on their toxicological profile.

**Results:**

In simulations, JWQS and MWQS had over 80% accuracy in classifying exposures as either strongly or weakly contributing to an association. In real data, both JWQS and MWQS consistently found that Pb and Cd exposure jointly predicted longer latency in the RVP and that second trimester exposure better predicted the results than the third trimester. Additionally, both JWQS and MWQS highlighted the strong association Cd and Sb had with lower accuracy in the RVP and that third trimester exposure was a better predictor than second trimester exposure.

**Conclusions:**

Our results indicate that metal mixtures effects vary across time, have distinct critical windows and that both JWQS and MWQS can determine longitudinal mixture effects including the cumulative contribution of each exposure and critical windows of effect.

## Background

Mixtures are the default human exposure scenario [1], but a major barrier in the investigation of mixture effects on health outcomes has been the lack of statistical tools that could address their impact and the variability of that impact both across the components of a mixture and across time. Because mixtures are the likely operational unit of a health effect, the study of single chemical exposures might reflect biased effect estimates on the health risks of the single chemical, as the actual effect is due to the mixture and is therefore contextual [2]. However, the impact of chemical mixtures on health may depend on the timing of exposure, i.e., critical windows of susceptibility [3, 4]. Additionally, the most relevant mixture may not be defined cross-sectionally but may instead reflect *joint exposure across time*, i.e., a multi-hit hypothesis [5]. Therefore, defining mixtures solely as cross-sectional combinations of joint exposure, even longitudinally, could miss mixture effects that occur across time. For example, chemical “a” measured in the second trimester of pregnancy might not interact with chemical “b” measured in the second trimester, but may instead interact with chemical “b” measured in the *third* trimester. This creates the need and opportunity to develop methods to better fit mixtures research into accepted biological theories such as developmental origins of health and disease (DOHaD), multi-hit hypotheses and critical windows of susceptibility. Prenatal exposures serve as a particularly important life stage to test such hypotheses, since this stage of development is characterized by a sequence of related, complex physiological changes that occur on a relatively short time-scale [6]. DOHaD, multi-hit hypotheses and critical windows all converge in prenatal life and uncovering periods of heightened windows of susceptibility is of great interest, particularly if they occur in a longitudinal, sequential manner.

Due to the difficulty in addressing both the relationships among multiple exposures occurring jointly and the temporal variability of mixture components, most statistical techniques have focused primarily on addressing multiple exposures at a single time point. Methods to assess the joint exposure-response relationships of multiple exposures at a single time point include: principal component analysis [7, 8], partial least squares regression [9], Bayesian kernel machine regression (BKMR) [10], classification and regression tree [11], k-means clustering [12], environmental risk score [13] and weighted quantile sum regression (WQS) [14]. While they do not address mixture effects across time, there are methods that study the time varying effect of a single pollutant across different time points, such as variations of distributed lag models (DLMs) [15–17]. There are two notable exceptions to this, the first of which involves the incorporation of a lag model into WQS regression called lagged WQS (LWQS) [3] and the second is a lagged version of BKMR, called *lagged* BKMR (LBKMR) [18]. Though these methods are, to our knowledge, the only ones that jointly address the issues of multiple correlated exposures across time, both have limitations. LWQS, through the use of a DLM, does not allow for the possibility of interactions between different exposures *across* different time points and the cross-sectional mixture effect. LBKMR is both computationally demanding and reliant on appropriate specification of distributions for the model parameters, thus its performance can suffer if the distribution of the data deviates significantly from the distribution implied by the model. In addition, due to the highly contextualized nature of the results, interpretation of LKMR results is not straight forward and can be difficult to explain succinctly.

To overcome these issues, we propose two new techniques, each leveraging the flexible WQS model but applying it differently in order to assess the impact of multiple exposures across different time points. The first method, called joint WQS (JWQS), consists of analyzing the set of exposures across time in a larger “exposure space,” thus allowing for relationships between different exposures across different time points. The second method is a hierarchical model called meta-WQS (MWQS) and consists of performing a WQS regression on each time point separately and then fitting a WQS model to the weighted combinations of exposures across time points. Both of these techniques are powerful ways to assess the association between repeated and complex combinations of exposures. We present both techniques in the same work to portray their complementary strengths and weaknesses. These strengths and weaknesses are also explored through simulations.

## Methods

### PROGRESS cohort description

Participants included in this analysis are mother and infant pairs enrolled in the *Programming Research in Obesity, Growth Environment and Social Stress* (PROGRESS) birth cohort study. Nine hundred forty eight pregnant women were recruited through the Mexican Social Security System (IMSS) from 2007 to 2011 and delivered a live infant. Women were eligible for the study if they were less than 20 weeks gestation, at least 18 years of age and planned to reside in Mexico City for the next 3 years. Women were excluded if they had a history of heart or kidney disease, consumed alcohol on a daily basis, used steroids or anti-epilepsy drugs or had a multiple gestation pregnancy. A more detailed description of the recruitment process can be found in detail elsewhere [19, 20]. The PROGRESS study protocols were approved by the institutional review boards (IRBs) of: the Icahn School of Medicine at Mount Sinai (IRB protocol number: 12–00751), Brigham and Women’s Hospital (IRB protocol number: 2006-P-001792) and the Mexican National Institute of Public Health (IRB protocol number: 560). Data from 393 mother-child pairs with an RVP assessment at 72 months of age, with complete data on blood metals and covariates, were selected for this work.

### Blood metal measurements

Prenatal metal exposure was determined using maternal blood samples drawn during the second trimester (between the 14th and 26th week of pregnancy) and third trimester (after the 26th week of pregnancy). Vacutainer tubes (Becton-Dickinson and Company, Franklin Lakes, New Jersey) were used to collect the samples, which were analyzed at the Lautenberg Laboratory for Environmental Health Sciences at Mount Sinai School of Medicine. Metal measurements were made using the Agilent 8800 Series triple-quadrupole inductively coupled plasma mass spectrometry coupled with high-performance liquid chromatography and a direct mercury analyzer.

### Rapid visual response

The measure of neurobehavior used in this study was the RVP, a test of executive function developed by Cambridge Neuropsychological Test Automated Battery (CANTAB, http://www.cambridgecognition.com/cantab). The test consists of a sequence of individual digits being presented to the participant who has to push a response pad when they detect a target sequence. The RVP task shares similarities with the Conner’s Continuous Performance Task (CCPT) [21], a test where decreases in performance have previously been linked to toxicant metal exposure [22–24]. The RVP measures several aspects of executive function, including: impulsivity, attention, working memory and processing speed. Two metrics of performance were used for this task: the overall percentage of responses that were correct (accuracy) and the median delay to a correct response (latency) to a correct response. Study personnel administering the test used a standardized protocol and were blind to the child’s level of metal exposure. Information on covariates includes: child sex, child age at RVP assessment, socio-economic status (SES), maternal education and maternal age at recruitment. The covariates were selected a priori. The SES index was determined using the 1994 Mexican Association of Intelligence Agencies Market and Opinion, which classified families into 6 levels based upon 13 questions regarding the characteristics of the household [25]. These 6 levels were collapsed into three SES tiers: low, medium and high.

### Weighted Quantile sum (WQS) regression

WQS was specifically developed to assess the joint effect of a combination of chemicals measured as a mixture. Applications have included metals and organics [26, 27]. Consider a set of *M* correlated metal concentrations at a single time point that are grouped into quantiles, *q*_*m*_, and an outcome of interest *y*. The general form of the weighted index model is

1 $$ E\left[y\right]\approx {\beta}_0+{\beta}_1\left(\sum \limits_{m=1}^M{w}_m{q}_m\right)+{\boldsymbol{z}}^{\mathrm{T}}\boldsymbol{\phi}, $$

where *β*_0_ is the intercept, *β*_1_ is the regression coefficient for the weighted sum of the quantiled metal concentrations and is constrained to be either nonpositive or nonnegative, ***z*** = [*z*_1_, …, *z*_*C*_] is the set of covariates, ***ϕ*** are the regression coefficients corresponding to ***z***, (∙)^T^ is the matrix transpose and *w*_*m*_ is the weight of the *m* th metal concentration, *q*_*m*_, which is constrained such that 0 ≤ *w*_*m*_ ≤ 1 and ${\sum}_{m=1}^M{w}_m=1$ [14].

In order to estimate generalizable *w*_*m*_, the data are first split into training and validation datasets; then *B* datasets are constructed using bootstrap samples drawn from the training dataset. For the results shown in this paper, the number of bootstrap datasets was 1000, the size of the training dataset is 40% of the subjects (approximately 157) and the size of the validation dataset is 60% of the subjects (approximately 236). All reported significances are those from the validation dataset. The proportions of the training/testing datasets are based upon those in [14]. Index weights are estimated for each bootstrap sample using maximum likelihood estimation in (1) and constrained such that the weights each must be both nonnegative and sum to one [14]. This means that the metals that have stronger associations with the outcome of interest for that particular bootstrap sample will have higher corresponding weights and those with less strong associations will have lower weights. The WQS index, *W*, is computed as:

2 $$ W=\sum \limits_{m=1}^M{\overline{w}}_m{q}_m, $$

where ${\overline{w}}_m=\frac{1}{B}\sum \limits_{b=1}^B{w}_{m,b}s\left({\hat{\beta}}_{1,b}\right)$, with *w*_*m*, *b*_ being the *m* th metal from the *b* th bootstrapped subsample and $s\left({\hat{\beta}}_{1,b}\right)$ is a measure of the significance of ${\hat{\beta}}_{1,b}$, or a measure of signal strength, in the *b* th subsample [14]. For this study, we use the value of the chi-square goodness-of-fit test as the measure of significance. Thus, combinations of metals with the strongest associations with the outcome will have higher relative values compared with combinations with lower associations. The overall significance of the WQS index can be determined by assessing the significance of the regression coefficient corresponding to *W*, *β*_WQS_, estimated from the following regression expression.

3 $$ E\left[{y}_v\right]\approx {\beta}_0+{\beta}_{\mathrm{WQS}}W+{\boldsymbol{z}}^{\mathrm{T}}\phi, $$

where *y*_*v*_ is the outcome data in the validation set [14]. All analyses in this study are adjusted for child age, child gender, SES, age of the mother at recruitment and maternal education. In order to test for a nonlinear relationship between the outcome and the derived WQS index, quadratic models of the following form are also assessed.

4 $$ E\left[{y}_v\right]\approx {\beta}_0+{\beta}_{{\mathrm{WQS}}_L}W+{\beta}_{{\mathrm{WQS}}_Q}{W}^2+{\boldsymbol{z}}^{\mathrm{T}}\phi . $$

### JWQS

JWQS extends WQS to the analysis of correlated exposures across time and enables the determination of the relative importance of exposures across time, thus providing information on critical windows for each exposure. Mechanistically, this is done by viewing the metal concentrations at different time points as exposures in a larger “exposure space” and performing WQS regression on this larger set of exposures. Thus, we use the term “joint WQS” analysis across both metals and time. This approach views time agnostically, meaning that there are no implicit constraints on the relationships between metal concentrations across time points as there are in a distributed lag model framework.

The JWQS framework for *M* metal concentrations at *T* time points is similar to the original WQS framework, with the following general model.

5 $$ E\left[y\right]\approx {\beta}_0+{\beta}_1\left(\sum \limits_{i=1}^{T\times M}{w}_i{q}_i\right)+{\boldsymbol{z}}^{\mathrm{T}}\phi, $$

where the weights, *w*_*i*_, are indexed across all combinations of metals and time points. Estimation of the indices and the corresponding regression coefficient proceeds as in the previous section. Note that, since the sum of the weights is equal to one, it is possible to determine the relative importance of the metals across time, by adding together their corresponding weights across all time points. Similarly, it is also possible to estimate the relative importance of the time points themselves with respect to the metal exposure, by adding together all weights corresponding to the metals at each time point.

### MWQS

The MWQS approach incorporates the temporal nature of metal mixtures using a two-stage, hierarchical approach. In the first part of the analysis, a WQS model is determined on the *M* metals at each time point, producing weights, *f*_*m*, *t*_, *m* = 1, …, *M*, *t* = 1…, *T*. Using these weights, a “total body burden index” [14], *v*_*t*_, for each time point, representing the total impact of all metals together on *y*, is calculated as

6 $$ {v}_t=\sum \limits_{m=1}^M{f}_{m,t}{q}_{m,t}. $$

Then, these WQS (total body burden) indices, derived for each time point, are grouped into quantiles, *p*_*t*_. Next, the final WQS model is estimated where the exposure are the quantiled total body burden indices for each time point, *p*_*t*_.

Due to the fact that the exposures in the final WQS analysis consist of the total body burden indices for each time point, the weights, *z*_*t*_, generated from this analysis determine the relative importance of each time point to the outcome *y*. Since these weights sum to one, they can be used to determine the relative importance of the *m* th metal at the *t* th time point, *w*_*m*, *t*_, with respect to each other as

7 $$ {w}_{m,t}={z}_t{f}_{m,t}. $$

This means that the relative importance of each metal across time points can be determined in a similar manner to JWQS by adding together the weights, *w*_*m*, *t*_, corresponding to the same metal across time points.

### Simulation setup

We use simulated datasets to demonstrate the application of the two proposed techniques and to compare their performances using a known ground truth. The simulated data are designed such that they have similar properties to PROGRESS data. This means that we modeled six exposures across two time points, i.e., *X*_*m*, *t*_, *m* = 1, …. , 6 and *t* = 1, 2. These exposures are generated according to

8 $$ \boldsymbol{X}=\left[{X}_{1,1}\dots {X}_{1,6}{X}_{2,1}\dots {X}_{6,2}\right]\sim N\left(\mathbf{0},\boldsymbol{C}\right), $$

where **0** ∈ *ℝ*^12^ and ***C*** is the estimated Pearson correlation matrix of the metals across time in the PROGRESS data. We then generate an artificial outcome as

9 $$ y=\boldsymbol{X}\mathbf{B} +\upvarepsilon, $$

where **Β** is a set of regression coefficients and *ε* is independent and identically distributed white Gaussian noise with zero mean and unit variance. The number of samples is equal to 393, the number of subjects in our study. We consider two scenarios, the first is when **Β** = [0, 0.1,0.2,0, 0, 0, 0,0.15,0.15,0, 0, 0]^*T*^, representing roughly equal weight between the two time points. The second is when **Β** = [0, 0.2,0.2,0, 0, 0, 0, 0, 0.15,0, 0, 0]^*T*^ and represents less equal weight between the two time points. Note that based upon [8], the first six elements of **Β** correspond to the effects of the exposures from the first time point and the second six elements correspond to the effects of the exposures from the second time point. These effects are similar to those observed in the PROGRESS data.

In these simulations, we generate 100 independent datasets and apply both JWQS and MWQS. We measure their performance using three metrics: accuracy, sensitivity and specificity. The WQS model designates an exposure as a significant contributor if the weight corresponding to that exposure is greater than 1 divided by the total number of exposures across time points, i.e., 1/12. We define accuracy as fraction of total weights, 12, where a modeled contributor is declared to be a contributor and a modeled non-contributor was declared to be a non-contributor. For example, the second, third, eighth and ninth exposures in scenario 1 are modeled contributors and the second, third and ninth exposures in scenario 2 are modeled contributors. We define sensitivity as the fraction of total modeled contributors that were declared to be contributors. Similarly, we define specificity as the fraction of total modeled non-contributors that were declared to be non-contributors. We report the results below.

### Data screening and analysis

The exposure data were grouped into deciles. Then, the data were split into a training set of 40% (approximately 157 subjects depending on the seed) and a testing set of 60% (approximately 236 subjects depending on the seed). All reported statistics and test-fit criteria are from the testing set. For both JWQS and MWQS, three different seeds for the assignment of subjects as being a part of the training set or validation set were used and the results of the models with the median Bayesian information criterion (BIC) were used. Linear models were compared with quadratic models and the model with the lower BIC value was used. In the derivation of the indices, the models were constrained to be either nonnegative or nonpositive. Models where the constraints were reversed were found to not be significant at a threshold of *p* < 0.1, so are not reported. Both outcome variables were log-transformed and treated as Gaussian distributed in the analyses. All analyses were performed using SAS 9.4.

## Results

### Simulation results

The results of the simulation study are summarized in Table 1. Overall both methods perform well in both scenarios, with an overall accuracy of at least 83.2% for both methods in the two scenarios. We find that both JWQS and MWQS perform better in terms of accuracy when the two time points have similar contributions (scenario 1). We also find that both methods have a higher specificity in scenario 1 compared to scenario 2 and a higher sensitivity in scenario 2 compared to scenario 1. This may be due to a combination of two factors. The first is the fact that the amount of collinearity is reduced in scenario 2, making it easier to identify the true contributors. The second is the exposures are less directly related to the outcome, due to the fact that only three elements of **Β** are non-zero in scenario 2 compared to four in scenario 1. This means that the line between contributors and non-contributors may become less well-defined and both methods are over-estimating the number of contributors.

**Table 1:** Simulation Results. Mean (standard error) performance for JWQS and MWQS for two scenarios

	JWQS			MWQS		
	Accuracy	Sensitivity	Specificity	Accuracy	Sensitivity	Specificity
Scenario 1	85.4 (0.1)	82.8 (1.3)	86.7 (1.0)	86.7 (1.3)	83.1 (2.2)	88.5 (1.2)
Scenario 2	83.2 (1.1)	90.3 (1.1)	80.9 (1.3)	83.2 (1.3)	86.7 (1.8)	82.1 (1.4)

#### Results for the analysis of PROGRESS data

**Fig. 1:**
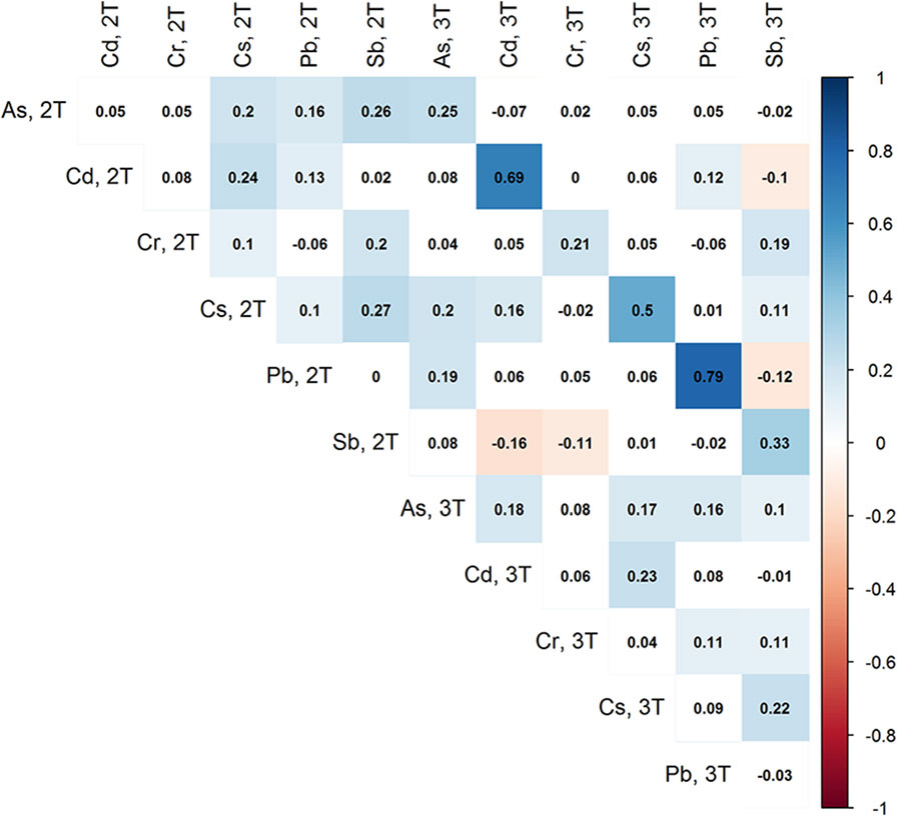
Spearman correlation between metals measured during the second trimester, 2 T, and third trimester, 3 T. Higher positive correlations are represented in darker blue and higher negative correlations are represented by darker red. Boxes without any color were not significant at a threshold of *p* < 0.05

Of the 948 subjects initially enrolled, 602 returned when their children were approximately 7 years old and 393 of these subjects had complete data on exposure, phenotype and covariates. The demographics of this subset are presented in Table 2 and the estimated metals concentrations are shown in Table 3. Figure 1 shows the Spearman correlation between each of the metals across the two time points. We see that the correlation structure is complex but that that there is a high correlation for the same metal measured at the two different time points. The average age of the children was 6.7 years and ranged from 6 years to 9 years. The average age of the women when they were recruited was 27.9 years but ranged from 18 to 44 years of age. Most of the women had more than a high school education (59%), but more than half (53%) were of low SES. Maternal age and maternal SES did not significantly differ between the subset of subjects included in these analyses and those in the parent cohort. There were differences between level of maternal education and child sex for the participants in our analyses and those in the entire parent cohort. We adjusted for these variables in our analyses.

**Table 2:** Demographic information and descriptive statistics

Demographics	Category	N (%)
Total		393 (100)
Child gender	Female	196 (50)
	Male	197 (50)
Socioeconomic status	Low	207 (53)
	Medium or high	186 (47)
Maternal education	Less than high school	163 (41)
	More than high school	230 (59)
		Average + SD (range)
Maternal age at recruitment (years)		27.9 + 5.5 (18.0–44.0)
Child age at RVP assessment (years)		6.7 + 0.6 (6.0–9.7)
RVP: delay to correct response (milliseconds)		744 + 262 (199–1670)
RVP: percent correct (%)		30.0 + 19.5 (3.7–92.5)

*SD* Standard deviation

**Table 3:** Blood metal levels measured in women during their second and third trimesters of pregnancy

Time Point	Metal	Average + SD (range)
Second Trimester	As (μg/L)	0.92 + 1.46 (0.28–27.52)
	Cd (μg/L)	0.29 + 0.20 (0.06–1.55)
	Cr (μg/L)	0.80 + 1.33 (0.00–1.43)
	Cs (μg/L)	3.14 + 1.34 (0.34–10.76)
	Pb (μg/dL)	3.68 + 2.59 (0.84–17.81)
	Sb (μg/L)	3.83 + 1.09 (1.79–11.25)
Third Trimester	As (μg/L)	0.94 + 0.74 (0.27–7.31)
	Cd (μg/L)	0.27 + 0.17 (0.05–1.77)
	Cr (μg/L)	1.12 + 3.12 (0.03–43.98)
	Cs (μg/L)	2.78 + 1.33 (0.15–9.46)
	Pb (μg/dL)	3.83 + 2.88 (0.02–28.25)
	Sb (μg/L)	3.94 + 0.96 (1.94–10.81)

*SD* Standard deviation

**Fig. 2:**
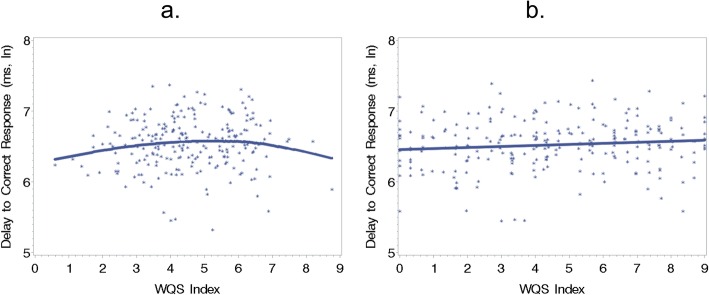
Plot showing the relationship between RVP latency and the (**a**) JWQS and (**b**) MWQS indices. Both plots are locally estimated scatterplot smoothing (LOESS) plots. The individual points in both plots are residuals accounting for the covariates: child age, child gender, maternal socio-economic status and age of the mother at recruitment. The WQS index estimated using JWQS was significantly and nonlinearly associated with the latency in the RVP (*β* [95%CI], *p*-value: 0.19 [0.03, 0.35], *p* < 0.05) for the linear term and (− 0.02 [− 0.04, − 0.00], *p* < 0.05) for the quadratic term. The WQS index estimated using MWQS was marginally linearly associated with the latency in the RVP (0.02 [− 0.00, 0.03], *p* < 0.10). Ln: Natural log

In the covariate-adjusted JWQS model, an index of metals across time points was found to be significantly and nonlinearly (quadratic) associated with the median latency time in the RVP, (*β* [95%CI], *p*-value: 0.19 [0.03, 0.35], *p* < 0.05) for the linear term and (− 0.02 [− 0.04, − 0.00], *p* < 0.05) for the quadratic term (Fig. 2a). This model had a Bayesian information criterion (BIC) value of 220. The linear term in the JWQS model was constrained to be nonnegative, indicating a longer time to a correct response is associated with an increasing value of the index. This model could be interpreted as increasing exposure is associated with worse performance (i.e., a greater delay to a correct response), but this association is attenuated for higher levels of exposure. This attenuation may be due to the few samples that exist for larger values of the index. Models where the direction of the association was constrained to be nonpositive were also generated, but these models were not significant at a threshold of *p* < 0.10, so we exclude them from the results.

**Fig. 3:**
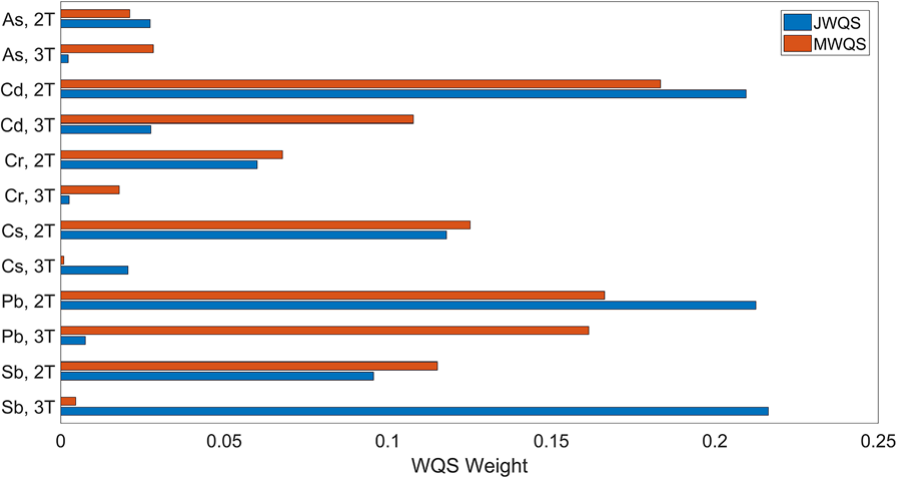
Bar graph of metal/time index weights for the indices associated with latency in the RVP. The two time points are the second trimester, 2 T, and third trimester, 3 T. Larger weights indicate greater impact of metals at individual time points. JWQS assesses the entire exposure space through the performance of a single WQS analysis. MWQS is a hierarchical model consisting of multiple WQS analyses first within a time point, then across time points

Figure 3 shows the contribution of different metals at different time points to the index. The top three weights correspond to third trimester Sb (21.6%), second trimester Pb (21.2%) and second trimester Cd (21.0%). Together these metals contribute 63.8% of the total weight to the index. Assessing the cumulative impact of different metals across time by adding together the weight of the same metal at the second and third trimesters, we find that the three metals with the highest overall weight over time are: Sb (31.1%), Cd (23.7%) and Pb (22.0%). Together these metals represent 76.8% of the total weight.

Assessing the overall impact of different time points by adding the weight for all metals within the time point (Table 4), we find that the second trimester contributes 72.3% of the total weight to the index, while third trimester metals only contribute 27.7% of the total weight.

**Table 4:** Index weights of time points for JWQS and MWQS for both RVP performance metrics (*n* = 393)

	Delay to Correct Response		Percent Correct	
	JWQS	MWQS	JWQS	MWQS
Second Trimester	72.3	67.9	45.5	36.0
Third Trimester	27.7	32.1	54.5	64.0

Larger weights indicate greater impact of individual time points across all metals. The weight for an individual time point is computed by adding together the WQS weights of each metal at that time point. JWQS assess the entire exposure space through the performance of a single WQS analysis. MWQS is a hierarchical model consisting of multiple WQS analyses first within a time point, then across time points.

In the covariate-adjusted MWQS model shown in Fig. 2b, we found a marginal, positive association between an index of metals across time points and the median latency in the RVP, (0.02 [− 0.00, 0.03], *p* < 0.10). This model had a BIC value of 215. In this model, the linear term was constrained to be nonnegative, indicating that higher values of the index are associated with greater latency to a correct response. We note that the results found when the linear term was constrained to be nonpositive were not significant at a threshold of *p* < 0.10, so we exclude them from the results.

In Fig. 3, we show the contribution of different metals at different time points to the index. The top three weights correspond to second trimester Cd (18.3%), second trimester Pb (16.6%) and third trimester Pb (16.1%). The weight to the index represented by these three exposures together was 51.0% of the total weight in the index. For this index, the three metals with the highest overall weight across time are: Pb (32.8%), Cd (29.1%) and Cs (12.6%). Together these metals represent 74.5% of the total weight in the index.

We also assess the overall impact of different time points and show these results in Table 4. We find that second trimester metals contribute 67.9% of the total weight to the index, while third trimester metals only contribute 32.1% of the total weight.

**Fig. 4:**
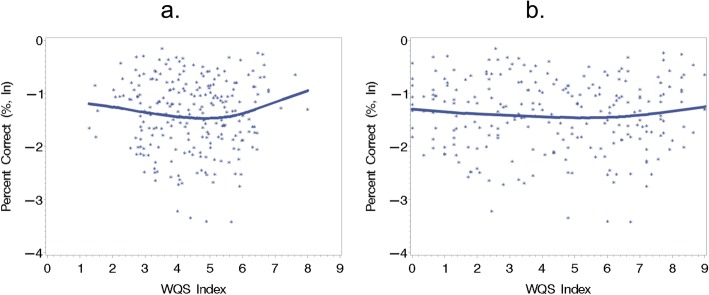
Plot showing the relationship between RVP accuracy and the (**a**) JWQS and (**b**) MWQS indices. Both plots are locally estimated scatterplot smoothing (LOESS) plots. The individual points in both plots are residuals accounting for the covariates: child age, child gender, maternal socio-economic status and age of the mother at recruitment. The WQS index estimated using JWQS was significantly and nonlinearly associated with the accuracy in the RVP (*β* [95%CI], *p*-value: − 0.41 [− 0.79, − 0.03], *p* < 0.05) for the linear term and (0.05 [0.00, 0.09], *p* < 0.05). The WQS index estimated using MWQS was marginally and nonlinearly associated with the accuracy in the RVP (− 0.12 ([− 0.26, 0.01], *p* < 0.10) for the linear term and (0.01 [− 0.00, 0.03], *p* < 0.10) for the quadratic term. Ln: Natural log

We find that an index of metals across time points was found to be significantly and nonlinearly (quadratic) associated with the accuracy in the RVP, (− 0.41 [− 0.79, − 0.03], *p* < 0.05) for the linear term and (0.05 [0.00, 0.09], *p* < 0.05) for the quadratic term, after adjusting for covariates (Fig. 4a). This model had a BIC value of 537. In this model, the linear term in the JWQS model was constrained to be nonpositive, indicating that an increase in the index corresponded to a decrease in the probability of a correct response. In this model, increasing values of the index are associated with a lower probability of a correct response, but this association is attenuated for higher levels of exposure. This attenuation may result from the few samples that exist for high values of the index. The results were not significant at a threshold of *p* < 0.10 when the linear term was constrained to be nonnegative, so they are excluded them from the results.

**Fig. 5:**
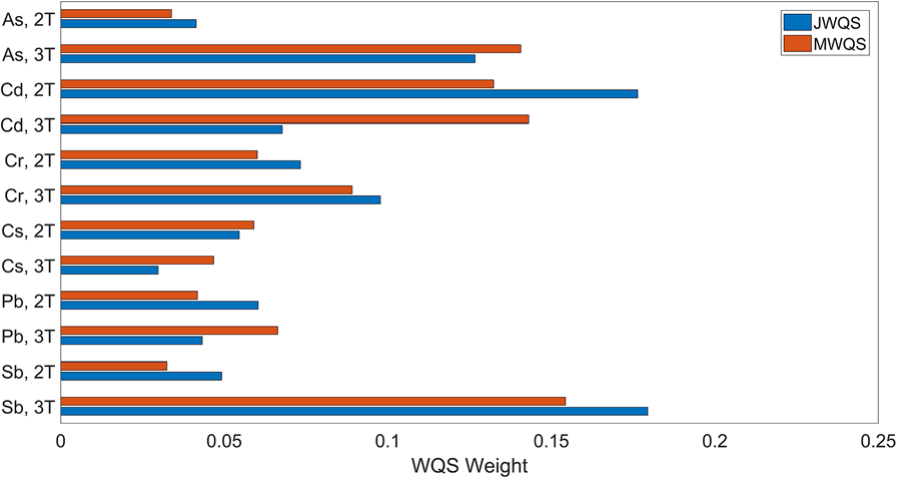
Bar graph of metal/time index weights for the indices associated with accuracy in the RVP. The two time points are the second trimester, 2 T, and third trimester, 3 T. Larger weights indicate greater impact of metals at individual time points. JWQS assesses the entire exposure space through the performance of a single WQS analysis. MWQS is a hierarchical model consisting of multiple WQS analyses first within a time point, then across time points

Figure 5 shows the contribution of different metals at different time points to the index. The top three weights correspond to third trimester Sb (18.0%), second trimester Cd (17.6%) and second trimester As (12.7%). Together these metals contribute 43.8% of the total weight to the index. Assessing the cumulative impact of different metals across time, we find that the three metals with the highest overall weight over time are: Cd (24.4%), Sb (22.9%) and Cr (17.1%). Together these metals represent 64.4% of the total weight.

We determine the overall impact of different time points in Table 4**.** We find that the second trimester contributes 45.5% of the total weight to the index, while third trimester metals contribute 54.5% of the total weight.

In the covariate-adjusted MWQS model shown in Fig. 4b, an index of metals across time points was found to be marginally significantly and nonlinearly (quadratic) associated with the accuracy in the RVP, (− 0.12 ([− 0.26,0.01], *p* < 0.10) for the linear term and (0.01 [− 0.00 0.03], *p* < 0.10) for the quadratic term. This model had a BIC value of 538. The linear term in the MWQS model was constrained to be nonpositive, indicating that an increase in the index is associated with lower accuracy. The interpretation of this model is that increasing values of the index are associated with lower accuracy, but this association is partially attenuated for higher levels of exposure. Results when the linear term was constrained to be nonnegative were not significant at a threshold of *p* < 0.10, therefore we exclude them from the results. The top three weights correspond to third trimester Sb (15.4%), third trimester Cd (14.3%) and third trimester As (14.1%) (Fig. 5). Together these metals contribute 53.8% of the total weight to the index. For this index, the three metals with the highest overall weight over time are: Cd (27.5%), Sb (18.7%) and As (17.5%). Together these metals represent 63.7% of the total weight.

Assessing the overall impact of different time points (Table 4), we find that second trimester metals contribute 36.0% of the total weight to the index, while third trimester metals contribute 64.0% of the total weight.

## Discussion

We developed two novel methods, MWQS and JWQS, as extensions of the WQS model for mixtures that address mixture effects arising across different times of exposure, but also maintain the ability to address cumulative mixture effects including concurrent exposures. The flexibility of these approaches enables the identification of mixture effects that arise across time without making assumptions about which combinations of chemicals drive the effects. As these methods are not based on a priori hypotheses, we acknowledge that our findings should be replicated in independent populations. Nevertheless, we believe these methods have substantial value helping researchers in observational studies identify the most relevant exposure timing for chemical mixtures, particularly those assessed longitudinally. The methods also identify which combinations of chemicals are more likely to be active biologically in driving longitudinal mixture effects. We have demonstrated the utility of both proposed methods in simulation studies and their ability to determine the effects of prenatal exposure to six metals, As, Cd, Cr, Cs, Pb and Sb, measured at two different time points on both reaction time and accuracy on the RVP task, a test of attention, working memory and processing speed in school age children. In addition to demonstrating the utility of the MWQS and JWQS, to our knowledge, this study is the first to prospectively link fetal exposure to mixtures of environmental metals with executive function measures in the RVP six to 7 years later. The metals used to define our mixture were selected a priori due to their role as toxicants [28–30].

In our study, Pb and Cd were among the largest contributors to the JWQS and MWQS indices associated with the delay to the correct response in the RVP. We also found that Cd and Sb were among the largest contributors to the JWQS and MWQS indices associated with accuracy in the RVP. Though there is a limited literature studying the effects of environmental metal exposure on subsequent performance on the RVP, there has been research into the effect of metal exposure on intelligence, attention and hyperactivity. Attention is a core component of the RVP and a major contributor to both performance metrics. Cd, Pb and Sb exposure have all been associated with lower cognitive function [31–39] and attention problems, including: inattention, hyperactivity/impulsivity and risk of attention deficit hyperactivity disorder [40–45]. However, these studies assessed the associations for each metal independently and not as a mixture, meaning that the risks may be underestimated depending on the relative exposure levels of the three elements in the general population [2]. Underestimation of risk may be particularly problematic among at risk populations, which often have both higher levels of toxic exposures and higher levels of learning problems [46, 47].

Prenatal Pb and Cd exposures have been shown to have a synergistic and negative impact on subsequent cognitive function [48]. Though there is a lack of literature highlighting a synergistic effect of Sb and Cd on subsequent neurodevelopment, both metals have been shown to damage the hippocampus [49–52]. The hippocampus is linked to impulse control [53], attention and memory [54]. Therefore, we postulate that the effect of prenatal exposure to a mixture of metals, including Cd and Sb, on performance on the RVP might be related to damage to the hippocampus.

Cd, Pb and Sb are all able to cross the blood brain barrier (BBB) and also increase its permeability, which may indirectly facilitate further neurological damage from other exposures normally kept distinct from the central nervous system [55–57]. Cd and Pb have been shown to act synergistically as a mixture to disrupt the expression of glial fibrillary acidic protein, a crucial macromolecule in the BBB [58]. In rats, the effects of the combination of Cd and Pb have been shown to be timing dependent, with greater neurological defects in pups compared with adult rats [56]. This may be also due to the fragility of developing cerebral vessels being greater in pups when compared with the developed ones in adults [59]. Therefore, understanding the impact of early life toxic metal mixtures, particularly in utero mixture exposures, is of critical importance. Mechanistically, these metals may act indirectly by the generation of reactive oxygen species outside the central nervous system (CNS), which then exert effects on the CNS [36, 51, 60]. However, other potential pathways may exist, including: disrupting neurotransmitters, interacting with essential metals and stimulating enhanced apoptosis of certain brain structures [4, 61]. More in vivo animal studies, particularly using mixtures involving Sb, are needed in order to confirm these proposed pathways or discover new ones. A strength of conducting mixtures research in observational studies is that the work can inform the timing and dose of animal studies that seek to replicate the findings.

We present the two proposed WQS-based techniques in the same work in order to portray their complementary strengths and weaknesses. By modeling the entire exposure space jointly, JWQS can model any additive linear relationship between pairs of metals, including the same metal, across time points. This complex modeling capability comes at the cost of requiring a sufficiently high number of samples in order to derive generalizable estimates. Additionally, the performance of JWQS may suffer when the correlation between the exposures across time is high. On the other hand, since MWQS is a hierarchical model, it does not require as many parameters to be estimated at one time, meaning that it is useful when the number of samples is restricted or when there is high correlation between the same exposures across time points. However, the use of a hierarchical model restricts the ways in which metals can be related across time points and limits the amount of correlated information that is used. Also, any errors in the estimation of the relative weights of the predictors in the first stage will be propagated through the second stage of the model. Additionally, in the case where strong timing dependent effects are expected, the initial summarization within one time point may be too extreme. These issues may contribute to the overall lower significance in the models produced using MWQS than those of JWQS, though we note that they have comparable performance in the simulations.

Despite these differences, there is a strong degree of similarity between the results obtained in this application of JWQS and MWQS. Both Pb and Cd are found to be within the top three contributing metals to the JWQS and MWQS indices for the delay to the correct response in the RVP. Pb and Cd have both been previously found to negatively impact reaction time in both adults as well as children individually; however, these effects were not studied in the context of mixtures of metals [23, 34, 37, 62–64]. Additionally, JWQS and MWQS highlight the second trimester as the time point that has much greater impact on the delay to the correct response than the third trimester, since approximately 70% of the weight is derived from second trimester metals for both methods.

Similarly, for the accuracy in the RVP, we find that both JWQS and MWQS highlight Cd and Sb as two of the top three metals in terms of their cumulative effect. To our knowledge, this work is the first to highlight the connection between prenatal Sb exposure and subsequent neurodevelopment. We also find that both methods highlight third trimester exposure as more impactful than second trimester exposure; however the values of the weights across time points are much closer in value to one another, around 60% for the two methods, than for the indices constructed for the latency in the RVP. The fact that the weights for the two trimesters are more similar than they were for the indices associated with latency may explain why the weights across time points shown in Fig. 5 align more closely between JWQS and MWQS for this outcome. Since each time point contributes similarly to the overall indices, there is less of information lost in the first stage of MWQS.

Our study has multiple strengths. We develop two novel methods to assess mixture effects of combinations of correlated metal concentrations across time points, the contributions of each metal, as well as discern time-dependent effects. We demonstrated the feasibility and high performance of the proposed methods in realistic data. We applied both methods to the same data from the PROGRESS cohort, showing the consistency of our models and finding general agreement between the two methods. Our sample size enables us to validate the generalizability of all of our constructed indices by testing their significance on a separate set of subjects within the PROGRESS cohort. However, our study also has a few limitations. We use maternal blood as a surrogate measure of prenatal exposure, which may not represent the true fetal exposure. Both proposed methods are based upon additivity but are nonetheless robust. For example, the weighted index and corresponding weights approximate an additive relationship where the interpretation of the weights is most accurate; however, in the presence of an interaction, the index is still a first-order approximation of the mixture effect. Because environmental exposures are generally low in most child populations, the overall interaction effect is generally modest due to the low scale of the exposure. So while both models are best under the assumption of additivity, the mixture effect can still be approximated by the weighted index even if the components combine with an interaction. Additionally, we only assess the impact of exposure to metals at two time points during the prenatal period, since the subjects were not recruited until their second trimester. An investigation of whether prenatal or postnatal heavy metal exposure contributes more to neurodevelopment is a promising direction for future research.

## Conclusions

We have proposed two new methods, JWQS and MWQS, to estimate the time-varying and cumulative effects of mixtures of correlated exposures over multiple time points and the contributions of each mixture component to the mixture effect. Despite the modeling differences, the results obtained by the two methods in both simulations and with real data are similar, thus strengthening our confidence in both methods. JWQS and MWQS share two of the three highest contributing metals for each of the two performance metrics, Pb and Cd for reaction time and Sb and Cd for accuracy. Additionally, both methods highlight similar relative importance in terms of the timing of exposure for both performance metrics. Specifically, both methods found that second trimester metal mixture exposure had greater impact on reaction time; while third trimester metal mixture exposure had greater impact on accuracy. Understanding the time-varying nature of environmental exposure mixtures can lead to a better understanding of their joint toxicity, thus facilitating more effective interventions, and both JWQS and MWQS are powerful techniques to provide these insights.

## Data Availability

The Programming Research in Obesity, Growth, Environment and Social Stressors (PROGRESS) data sharing protocol for researchers at Icahn School of Medicine at Mount Sinai (ISMMS) and collaborating institutions, Mexican National Institute of Public Health, Harvard University and Columbia University requires researchers to obtain the Collaborative Institutional Training Initiative (CITI) certificate for the online course Research Ethics and Compliance Training. The CITI certificate along with a resume should be sent to the ISMMS administrative manager and PROGRESS data analyst (Nia McRea). Additionally, a data request form must be completed and sent to the principal investigators of the PROGRESS study, Drs. Robert O. Wright, Mara Tellez-Rojo and Andrea Baccarelli, for review and approval. The data sharing protocol for researchers not at ISMMS or collaborating institutions requires the researcher to complete all of the above mentioned steps in addition to filling out a data use agreement form. When these requirements are met, researchers are given access to the PROGRESS data through a safe data sharing service.

## Abbreviations

As: Arsenic
BBB: Blood brain barrier
BIC: Bayesian information criterion
BKMR: Bayesian kernel machine regression
*CANTAB*: *Cambridge Neuropsychological Test Automated Battery*
CCPT: Conner’s Continuous Performance Task
Cd: Cadmium
CNS: Central nervous system
Cr: Chromium
Cs: Cesium
DLMs: Distributed lag models
DOHaD: Developmental origins of health and disease
IMSS: Mexican Social Security System
IRBs: Institutional review boards
JWQS: Joint weighted quantile sum regression
LBKMR: Lagged Bayesian kernel machine regression
Ln: Natural log
LOESS: Locally weighted scatterplot smoothing
LWQS: Lagged weighted quantile sum regression
MWQS: Meta-weighted quantile sum regression
Pb: Lead
PROGRESS: Programming Research in Obesity, GRowth, Environment and Social Stressors
RVP: Rapid Visual Processing
Sb: Antimony
SD: Standard deviation
SES: Socio-economic status
WQS: Weighted Quantile Sum
WQS: Weighted quantile sum regression
